# Survival differences of CIMP subtypes integrated with CNA information in human breast cancer

**DOI:** 10.18632/oncotarget.16178

**Published:** 2017-03-14

**Authors:** Huihan Wang, Weili Yan, Shumei Zhang, Yue Gu, Yihan Wang, Yanjun Wei, Hongbo Liu, Fang Wang, Qiong Wu, Yan Zhang

**Affiliations:** ^1^ College of Bioinformatics Science and Technology, Harbin Medical University, Harbin, 150081, China; ^2^ School of Life Science and Technology, State Key Laboratory of Urban Water Resource and Environment, Harbin Institute of Technology, Harbin, 150001, China

**Keywords:** CpG island methylator phenotype (CIMP), breast cancer, copy number alteration (CNA), prognosis, biomarker

## Abstract

CpG island methylator phenotype of breast cancer is associated with widespread aberrant methylation at specified CpG islands and distinct patient outcomes. However, the influence of copy number contributing to the prognosis of tumors with different CpG island methylator phenotypes is still unclear. We analyzed both genetic (copy number) and epigenetic alterations in 765 breast cancers from The Cancer Genome Atlas data portal and got a panel of 15 biomarkers for copy number and methylation status evaluation. The gene panel identified two groups corresponding to distinct copy number profiles. In status of mere-loss copy number, patients were faced with a greater risk if they presented a higher CpG islands methylation pattern in biomarker panels. But for samples presenting merely-gained copy number, higher methylation level of CpG islands was associated with improved viability. In all, the integration of copy number alteration and methylation information enhanced the classification power on prognosis. Moreover, we found the molecular subtypes of breast cancer presented different distributions in two CpG island methylation phenotypes. Generated by the same set of human methylation 450K data, additional copy number information could provide insights into survival prediction of cancers with less heterogeneity and might help to determine the biomarkers for diagnosis and treatment for breast cancer patients in a more personalized approach.

## INTRODUCTION

Breast cancer consists a large part of cancer-induced mortality in female. About 1.7 million women were diagnosed with breast cancer in 2012 worldwide, ranking the first in the cancer occurrences of female [1]. This heterogeneous malignancy have several molecular subtypes: HER2, luminal A, luminal B, basal-like, and normal-like, which are mainly characterized by pathologic features and clinical behaviors associated with mutations of special genes [2]. Though well-classified, those molecular-determined subgroups remain to be well investigated on their molecular foundations and biological heterogeneity. Accumulative genomic aberrations, including genetic and epigenetic modifications have a profound impact on the heterogeneity among those subtypes.

Alterations in tumor progression bring to substantial abnormalities in human neoplasia like global hypomethylation and promoter CpG island hypermethylation [3, 4]. Growing evidence suggested that methylation at cancer-specific loci characterized a special phenotype in tumors, and this phenotype is nominated as CpG island methylator phenotype (CIMP) [5]. Toyota et al. first proposed this term to describe a subset of colorectal tumors presenting cancer-specific methylation pattern, and recent studies have also proved the existence of CIMP in breast tumors is related to characteristics like metastatic behaviors and clinical outcomes [6, 7]. Rooted in methylome aberrations, breast CIMP is different from molecular subtypes based on gene expression profiles and even can account for some transcriptional diversity related to gene expression-based subtyping [8]. For example, basal-like tumors have lower methylation pattern while CIMP-high samples are consist of a large part of luminal B breast carcinomas, and these expression patterns might be regulated through methylation [7–10]. Moreover, numerous publications concentrating on breast cancer methylome have suggested the DNA methylation can be used as a robust biomarker for clinical prediction of breast cancer, promoting the research on mechanism of CIMP [11].

Copy number alteration (CNA), a simple form of chromosomal instability, mainly refers to the gain or loss of genomic contents in gene sequences contrasted with reference genome. Like MSI, CNA also function as a widely-used genomic feature to classify cancers [12]. With least alteration size of 50 bp, CNA can involve a part of a gene, whole part of a gene and even its neighbors, the recurrent amplifications of oncogenes and deletions of tumor suppressor genes can induce phenotypic consequences and different clinical outcomes in cancer [13–15]. For example, in HER2-overexpressing breast cancers, increased expression of ERBB2 gene is associated with 17q21 copy number gain. Recently, Tabarestani et al. carried out a study on single copy number alteration of several prognosis-related genes (like CCND1, TOP2A), and the results strengthened their prognostic values in breast cancer [16]. Based on the premise that both epigenetic and genetic changes can influence gene expression patterns and pathways, several reports have addressed the overlap of genomic variations between CNA and methylation in breast cancer [17–19]. Moreover, Noushmehr et al. found the CIMP-positive and CIMP-negative tumors presented distinct copy number profile in glioma, strongly suggesting a relationship might be shared between CIMP and CNA [7]. All these evidences suggests that CIMP classification power can be enhanced if other currently-used approach like CNA information is introduced [12].

To integrate copy number and methylation alterations, and also to reduce the tumor heterogeneity in a more cost-efficient way, Feber et al. found and validated that Infinium HumanMethylation450 BeadChips (450K) had potentials to detect not only aberrant CpG methylation loci, but also regions with abnormal CNA [20]. In this study, we aimed to figure out the survival difference between CIMP-H and CIMP-L samples in different copy number status using the 450 k array data from the Cancer Genome Atlas (TCGA). Our study made a stratification of 765 samples according to hypermethylated genes with abnormal CNA which were picked out by our criteria. The survival analysis found that CIMP-L type had a better survival significantly in CnLoss but worse tendency in CnGain samples. The results may enable a more precise evaluation of CIMP and a broader sight into the molecular basis of CIMP related to the genomic changes caused by copy number changes.

## RESULTS

### Copy number characteristics of breast cancers

**Figure 1:**
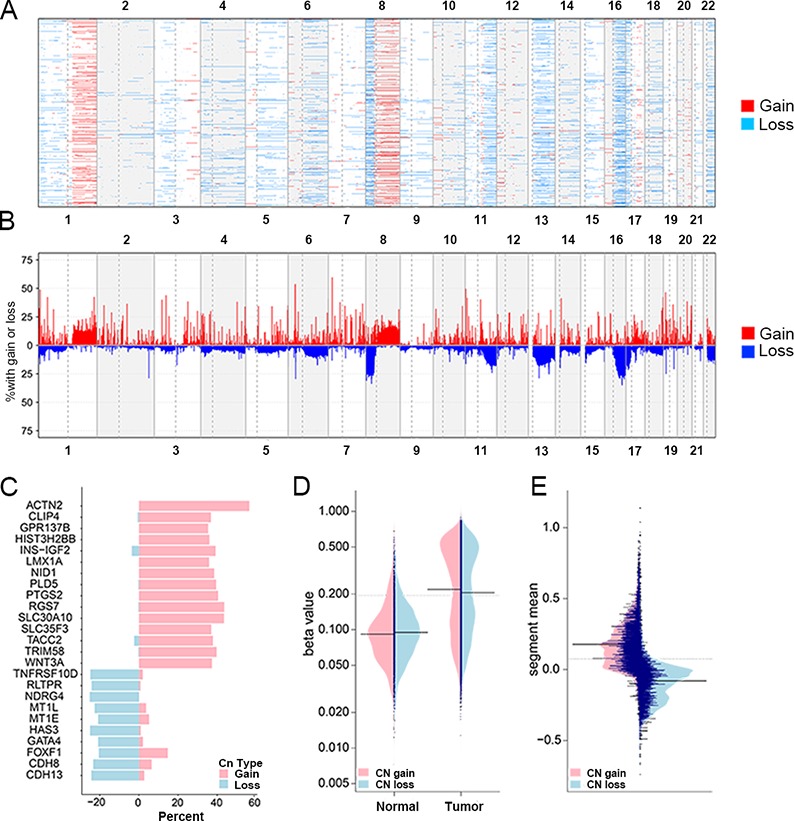
Copy number patterns of breast cancer genome and different methylation patterns of recurrent CNA genes Copy number alterations of the CG sites were analyzed with 765 breast cancers across 22 autosomes and were plotted in genomic coordinates along the x axis. Neutral/no change was indicated in white, gain was indicated in red and loss was indicated in dark red. **(A**) The copy number gain/loss occurrence in all tumors, the sites with segment mean > 0.3 were regarded as copy number gain, and < −0.2 for copy number loss (see materials and methods). (**B**) Total percentage of gain/loss is listed by the cumulative change per sample. **(C**) The copy number gain/loss recurrence of 25 markers (The percentage of occurrence in all tumors). (**D**) The methylation value distribution of genes in normal and tumor samples. The average methylation values of tumors was about 0.25, and the 3rd quantile was higher than 0.5. (**E**) The distribution of segment mean in CN gain and loss genes. The average segment mean of CN gain genes was about 0.2, and 0.1 in loss genes. The average changes of CN gain genes were larger than CN loss genes.

After quality control (see materials and methods), the analysis proceeded with 406584 probes and 861 samples. We checked the copy number profile and counted the cumulative percent of CNA on all chromosomes of all 765 tumors against the normal samples, and found the variations of genomic structures differed across those tumors (Figure 1A and 1B). The degree of copy number gain was larger than copy number loss, gains of chromosomes 1q and 8q and losses of chromosome 8p and 16q were identified, which is concordant with the genetic alterations of breast cancer [21, 22].

### Methylation patterns of markers with different copy number alteration

Most variant CpG loci in top 50 percent probes with most significant CV of methylation levels in tumors and intersect them by the genes with significant CNAs. We got 72 most variant probes of 25 genes, 10 presenting wide copy number loss and 15 copy number gain (see materials and methods). Most probes were located in CGIs of gene body and promoter (Supplementary Table 1). The locations of markers were listed in Table 1, most of genes with copy number gain are located in chromosome 1 and genes with copy number loss in chr16, which agreed with regions of significant copy number changes in previous studies on breast cancers [23, 24]. The recurrence percentage of CNA in those genes across all samples showed that two types of CNA (gain/loss) didn't co-occur, and CN gain genes apparently occurred more often than CN loss genes with higher extent in this dataset (Figure 1C). We also compared the methylation pattern of this gene panel in normal and tumor status using beanplot, a better alternative of boxplot for visual comparison of density distributions [25]. The average methylation level of all probes in normal sample cohorts was lower than the level in tumor cohorts (Figure 1D, left. *P* value < 2.2e-16, *t-test*). Likewise, in tumorous samples the methylation variances of those genes were significant. (Figure 1D, right. *P value* = 2.92e-15, *t-test*). The average methylation values of tumors was about 0.3, and the 3^rd^ quantile was lower than 0.5, here we chose 0.6 (0.45) as threshold to determine whether a CN loss (gain) gene was hypermethylated (Me (+)). According to the distribution of their segment means, the absolute segment mean values of CN gain genes were larger than CN loss gene, and 0.3 and −0.2 were used to determine whether a gene is gain (CN (+)) or loss (CN (−)) (Figure 1E). Moreover, we investigated the function of this marker panel using DAVID, and they mainly enriched in some causing-cancer biological processes, like regulation of cell-substrate adhesion, regulation of growth, negative regulation of cell communication (Supplementary Figure 1).

**Table 1:** The location of markers on genome and their dominant copy number status in most samples

chrom	start	end	geneid	symbol	CNA
1	165171103	165325952	1783	LMX1A	gain
1	186640943	186649559	1979	PTGS2	gain
1	220087605	220101993	2219	SLC30A10	gain
1	228194722	228248972	2293	WNT3A	gain
1	228645807	228646259	2309	HIST3H2BB	gain
1	234040678	234460262	2356	SLC35F3	gain
1	236139131	236228481	2375	NID1	gain
1	236305831	236372209	2376	GPR137B	gain
1	236849753	236927927	2382	ACTN2	gain
1	240938813	241520530	2395	RGS7	gain
1	242251688	242687998	2405	PLD5	gain
1	248020500	248043438	2452	TRIM58	gain
2	29320541	29406679	2706	CLIP4	gain
8	11561716	11617509	9971	GATA4	loss
8	22993100	23021543	10065	TNFRSF10D	loss
10	123748688	124014060	12689	TACC2	gain
11	2150341	2182439	12877	INS-IGF2	gain
16	56651372	56652730	18511	MT1L	loss
16	56659584	56661024	18512	MT1E	loss
16	58497548	58547523	18561	NDRG4	loss
16	61685914	62070739	18571	CDH8	loss
16	67679029	67691472	18624	RLTPR	loss
16	69139466	69152619	18659	HAS3	loss

### CNA and CIMP classification

**Figure 2:**
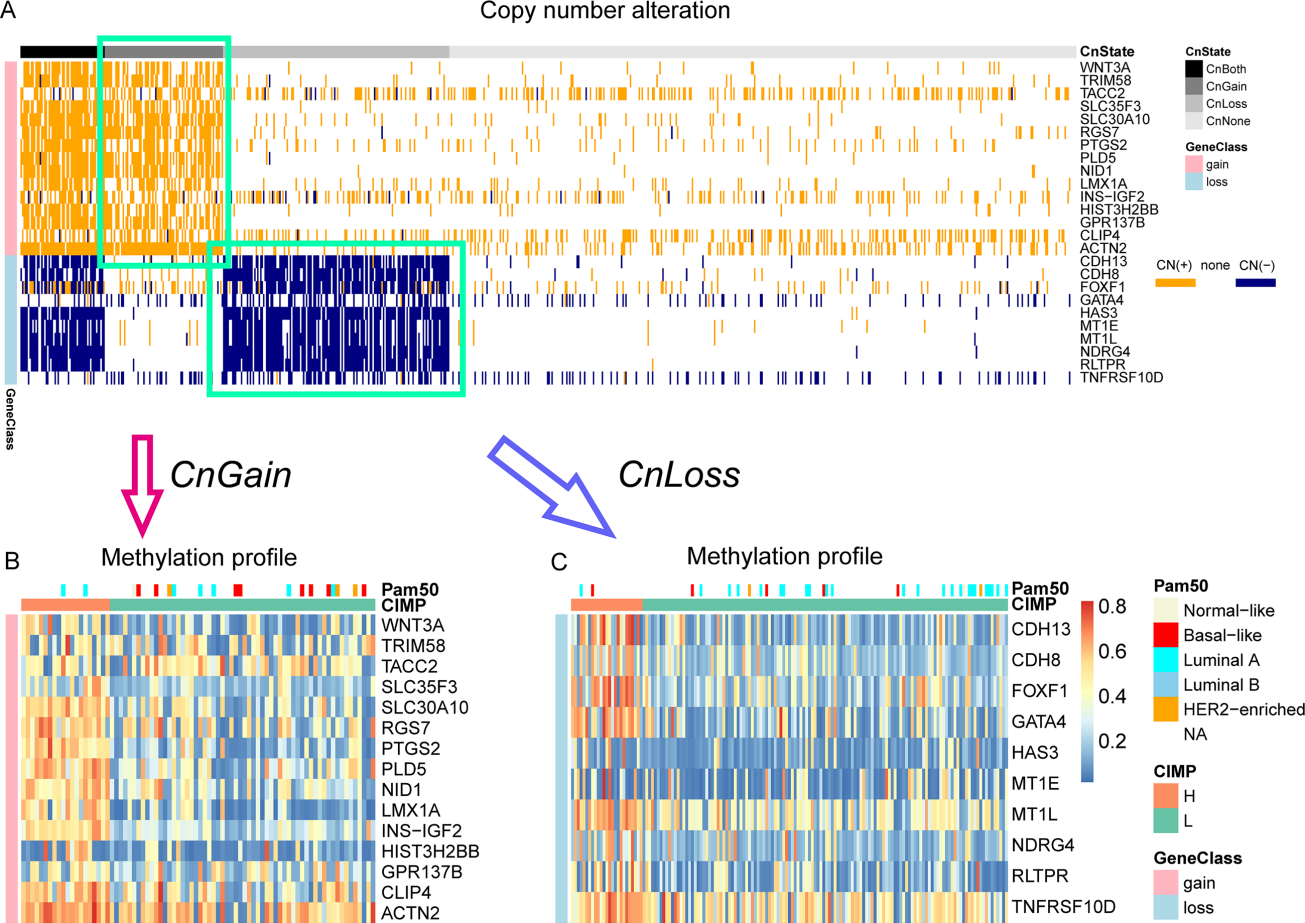
Heatmaps of binary copy number profile and methylation profile of CnGain/Loss samples (**A**) The binary copy number alteration profile. Samples were first classified into 4 copy number statuses by the number of CN (+) and CN (−) genes. CN (+): segment mean > 0.3; CN (−): segment mean < −0.2. (**B**) The methylation profile of CnGain samples. (**C**) The methylation profile of CnGain/Loss samples.

**Figure 3:**
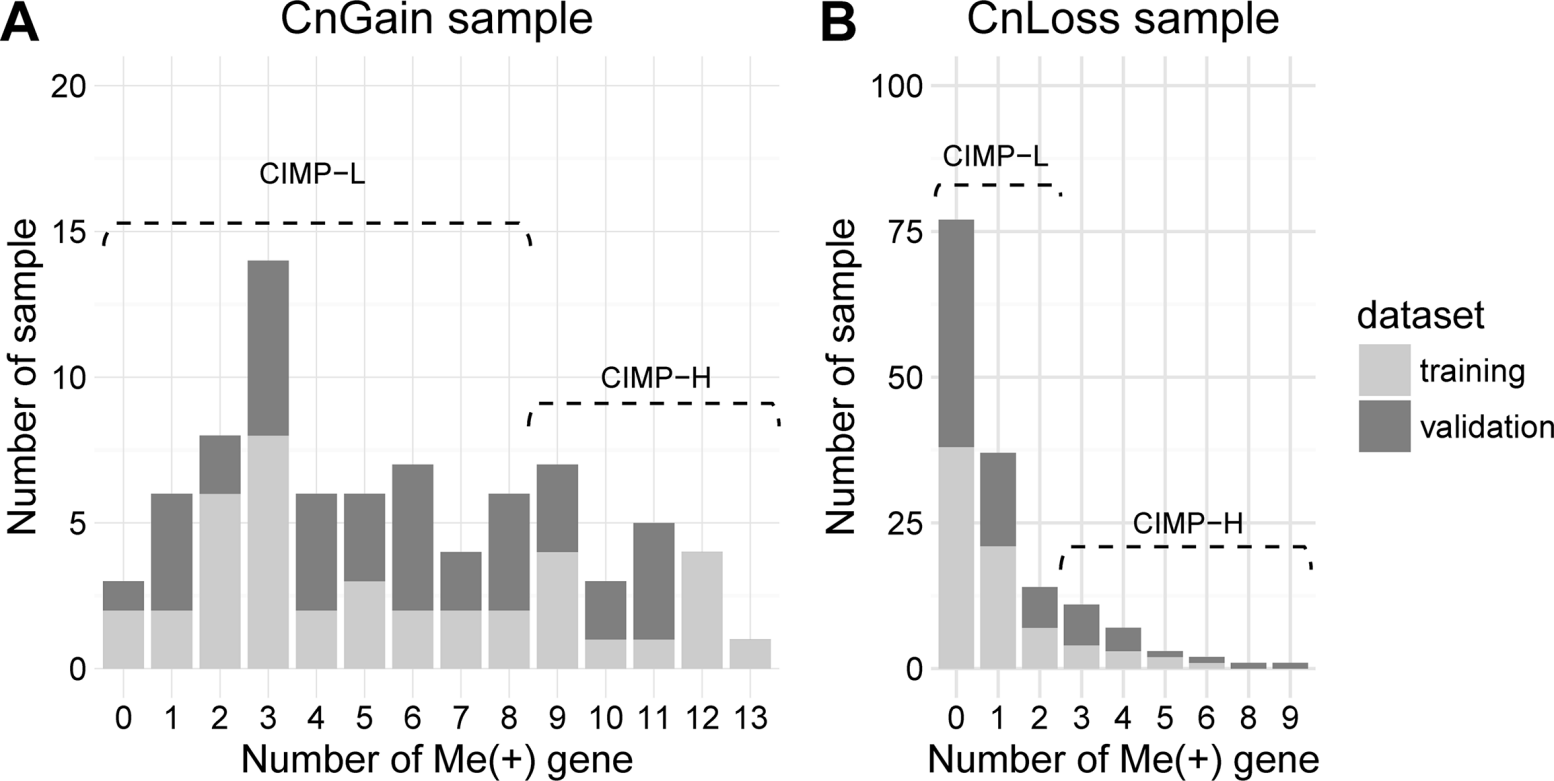
Enrichment analysis of PAM50 golden standard in the CIMP-H/L in CnGain/Loss samples * represents *p* < 0.05.

To merge the copy number information into CIMP stratification, the samples that tended to show either copy number gain only or loss only status were remained, which were referred as CnGain/Loss. To determine the copy number status of a sample, we counted CN (+) and CN (−) genes in our marker panel, and evaluated copy number status of a sample according to the percentage of CN (+/−) genes (See materials and methods). The copy number profile of samples that contained binary CN(+/−) information was presented in a heatmap (Figure 2A). Since it was hard to find out which type of CNA are dominant in the samples with both copy number gain and loss (CnBoth) for this dataset, those samples were removed. And samples with no alteration of copy number in our markers were also removed. So finally 80 CnGain samples with no less than 6/15 CN (+) genes and 153 CnLoss samples with no less than 3/10 CN (−) genes were remained. Then we analyzed the CIMP−H/L in CnLoss/Gain groups separately using CN loss/gain genes and found the methylation patterns of markers differed in CIMP-H/L in both copy number status (Figure 2B and 2C). Therefore, we counted the number of genes harboring hypermethylation in each CnGain/Loss sample and compared their cumulative frequency in all samples (Figure 3). It was evident that most samples presented low number of hypermethylated markers. Then CIMP-H was defined as having as least 9/15 Me (+) genes in CN gain genes, and this group constituting 25% (20/80) of CnGain samples (Figure 3A). As for CnLoss samples, CIMP-H samples were defined as having at least 3/10 Me (+) genes, making up 16.3% (25/153) in CnLoss group (Figure 3B).

Judged by the frequency distribution of Me (+) genes, most samples didn't present much hypermethylation measured by the gene panel, proving the strictness of our criteria on the other hand. Particularly, the ratio of CIMP-H/CIMP-L in CnGain status was larger than those of the CnLoss (25% and 16.3%). CIMP-H were much less than CIMP-L, which was consitent with the low fraction of CIMP-H in most studies researched on CIMP. The higher fraction of CIMP-H in CnGain samples indicated that for our gene panel, tumors with copy number gains might have high methylation levels and a relationship might be shared between CN loss and hypomethylation.

On the other hand, the distribution of clinical factors of all samples with the CnGain/Loss and CIMP-H/L was checked, but most of them were not significant, by Chi-square test (Supplementary Table 2). Particularly, among 233 CIMP samples in two copy number status, there were 69 samples with known PAM50 defined subtypes including Basal-like, HER2-enriched, Luminal A, Luminal B, and normal-like [26] (Supplementary Table 3). Basal-like and HER2-enriched subtypes appeared most frequently in CnGain/CIMP-L group, constituting 61.5% and 60% of their total amount. While CnLoss/CIMP-L contained the largest proportion of Luminal A cancers (74.2%), and CnLoss/CIMP-H contained the highest proportion of Luminal B with subtypes (38.9%). For the significance of the enrichment of PAM50 subtypes was determined by the hypergeometric test (Supplementary Figure 2). The CnLoss/CIMP-L group was significantly enriched in Luminal A subtype. While in CnGain/CIMP-L group, basal-like subtype tended to be a dominant part. CnLoss/CIMP-L was specially associated with Luminal B subtype. In brief, the enrichment difference of molecular subtypes may explain the heterogeneity existed in breast CIMP cancers to some degree.

**Figure 4:**
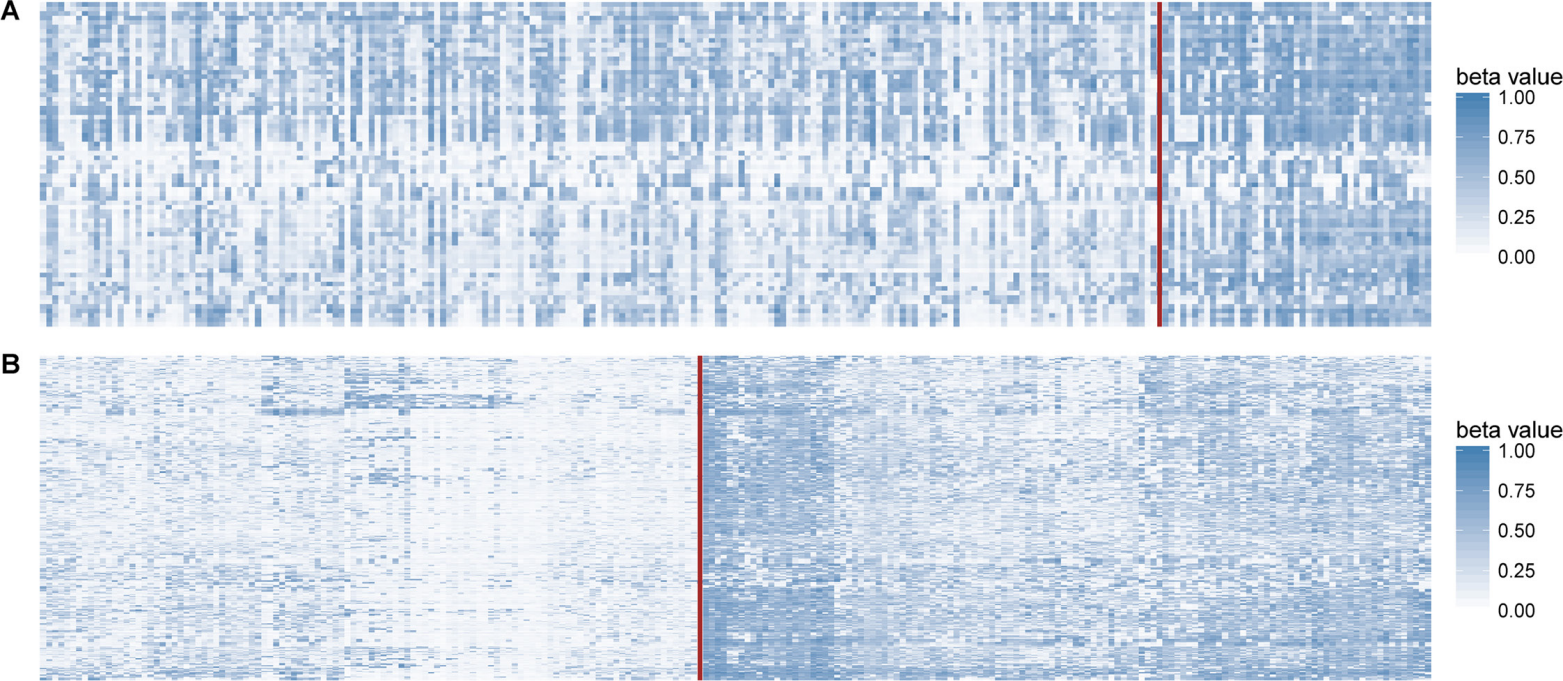
Methylation profiles of CIMP-H/L The order of samples in the heatmap followed the order of CIMP-L and CIMP-H, between which were separated by a red line. (**A**) Samples divided by our CIMP classification criteria were presented in the heat map. (**B**) Heat map created by hierarchical clustering of the same cohort but the labels were 601 differentially methylated probes with most 20% significant CV in DMGs. Samples in lower methylation status were in the left part of both heat maps. Concordant hypermethylation in a subset of tumors was observed by different classification methods, but clustering method yielded more CIMP-H samples.

To have a look at our CIMP subtypes in a more comparative way, we also visualized the methylation patterns of CIMP-H/L by hierarchical clustering and by our method (Figure 4). Similar to the results generated by hierarchical clustering method using 601 differentially methylated probes (Euclidean distance, Ward-D2 method), the samples also aggregated into two clusters using our classification rule. The results of hierarchical clustering yielded 49 CIMP-H in 80 CnGain samples and 73 CIMP-H in 153 CnLoss samples, with the overlap of 41 CIMP-H and 107 CIMP-L with our method. In summary, more CIMP-H samples were figured out by the clustering method than our method, which contradicted the phenomena that CIMP-H consisting less part of tumors than CIMP-L in most studies.

### Survival analysis of CIMP groups in different CNA status

**Figure 5:**
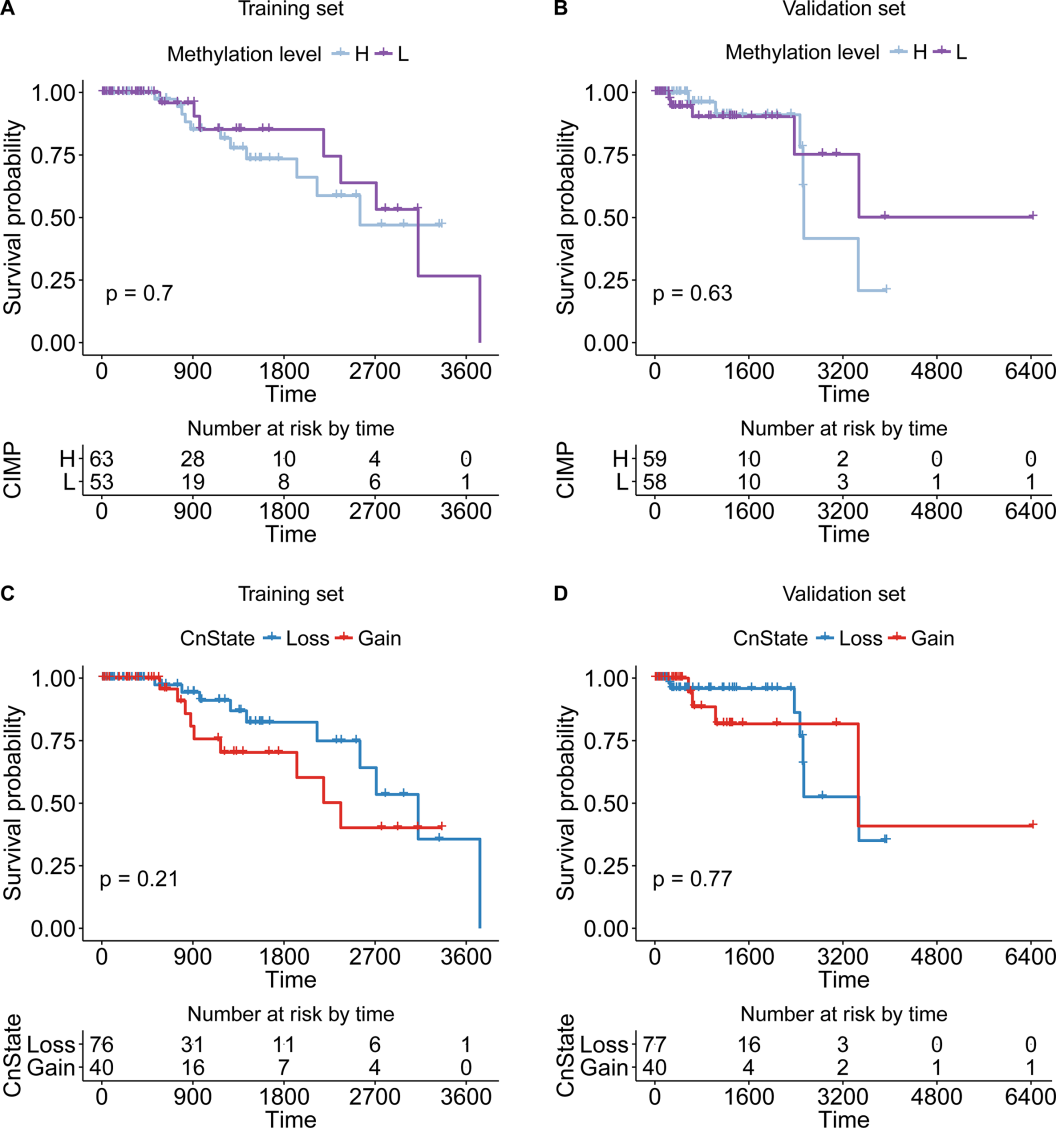
The KM plot of CIMP-H/L in CnLoss/Gain samples divided into training and validation set (**A**, **B**) The KM plot of CIMP-H and CIMP-L samples in CnLoss status, training/validation set. (**C**, **D**) The KM plot of CIMP-H and CIMP-L samples in CnGain status, training/validation set.

**Figure 6:**
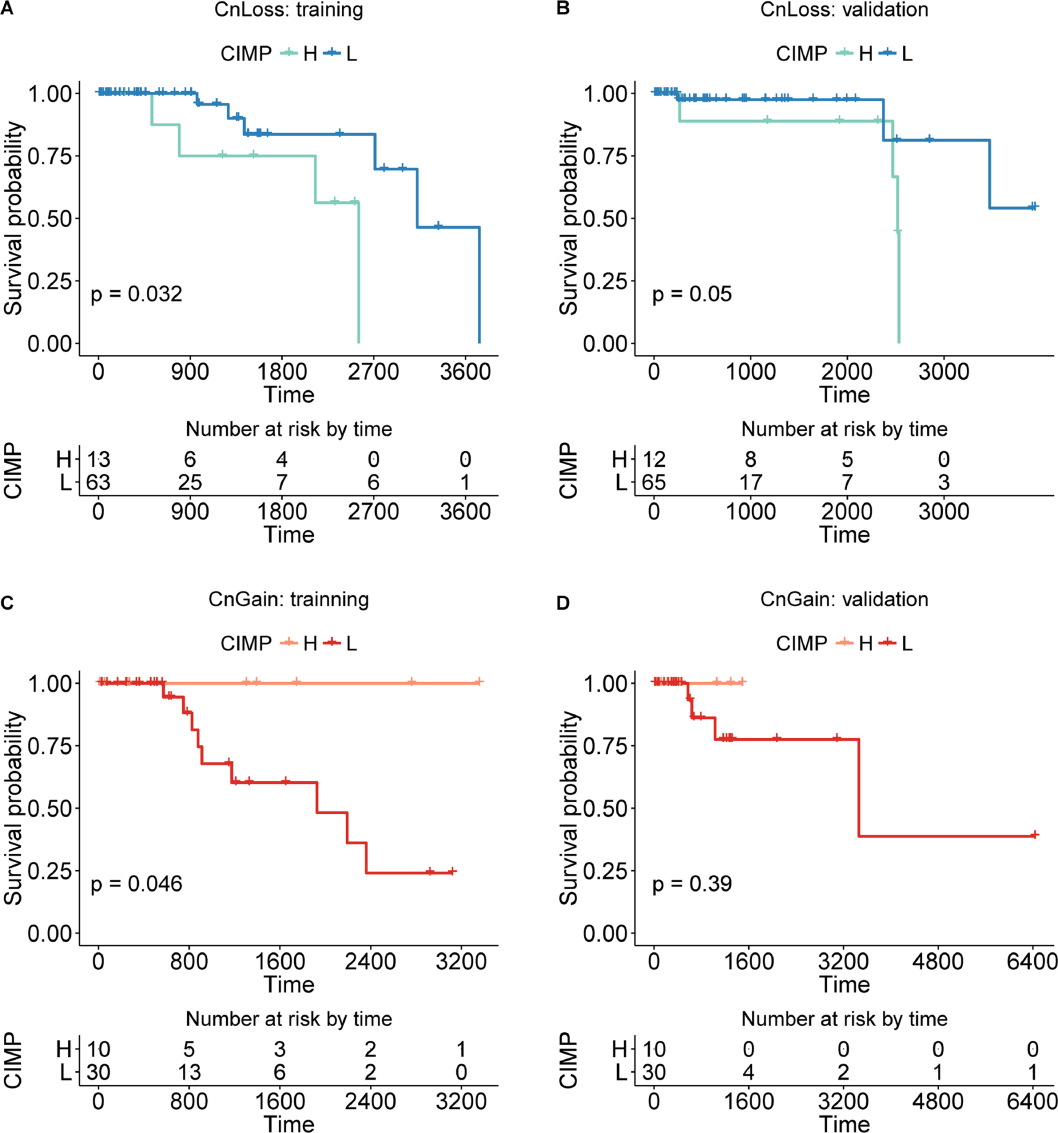
The KM plot of CIMP-H/L or CnLoss/Gain samples. The significance of two types of classification was not significant (**A**, **B**) The KM plot of CIMP-H/L classified by clustering method of training/validation set. (**C**, **D**) The KM plot of CnGain/Loss samples in training/validation set.

To highlight the power of our method stratifying CIMP based on copy number status of samples, we first checked the survival difference of CIMP-H/L classified by the clustering method without copy number information, which was detailed above (Figure 5A and 5C). And the survival difference of CnGain/Loss was also checked (Figure 5B and 5D). Both results of preliminary classifications just based on methylation or copy number information were not significant in training or validation set. However, the survival of CIMP-H/L varied significantly in CnLoss samples after adding the genomic information of copy number alteration (Figure 6). The KM plots were draw for both CN groups in training and validation sets. The univariate Cox proportional hazard regression model found that in CnLoss samples, CIMP-L outperformed CIMP-H (HR = 0. 219; 95% CI = 0.048–0.999; log-rank *P* = 0.032) in training set, and the difference is still significant in validation set (HR = 0.199; 95% CI = 0.034–1.151; log-rank *P* = 0.05). Inversely, the survival of CIMP-L was worse than CIMP-H in CnGain status in training set, but not significant in validation set. Several clinical factors like age, stage and menopause were also checked by multivariate Cox regression model, but most of them were not significant (Table 2, Supplementary Table 4).

**Table 2:** The univariate and multivariate Cox proportional hazard regression of CIMP-H/L and other clinical factors in training set

Clinical factors	Univariate		Multivariate	
	Hazard ratio (95% CI)	*P* value	Hazard ratio (95% CI)	*P* value
**Age(continuos)**	1.002 (0.954–1.053)	0.9362	0.943 (0.813–1.094)	0.4397
**Lymph nodes**	1.044 (0.961–1.134)	0.3075	97.409 (3.458–2744.295)	0.0072
**Stage**				
Stage I/II	ref		ref	
Stage III	2.174 (0.575–8.213)	0.2523	355.567 (0.892–1.4E4)	0.0545
Stage IV				
Stage X				
**Histological type**				
Infiltrating Ductal	ref		ref	
Infiltrating Lobular	1.017 (0.222–4.663)	0.983	13.808 (0.013–1.4E4)	0.458
Mixed histology	0.88 (0.092–8.401)	0.9112	286E7(117.613–6.93E+12)	0.0067
Other				
**Menopause**				
Interminate				
Peri	NA (NA–NA)		–	
Post	0.08 (0.007–0.903)	0.0411	461.861 (2.05–104038.129)	0.0264
Pre	0.039 (0.002–0.81)	0.036	–	
**CIMP in CNA–none**				
CIMP–L/CIMP–H	0.219 (0.048–0.999)	0.0499	0 (0–0.174)	0.0134

The hazard rations of some clinical factors were not calculated because the amount of samples in that group was not enough for the estimation of risk.

This result not only indicated that our method stood above the clustering method, but also validated our supposition that the CIMP classification could be better described when more genomic information integrated. Therefore, messages related to genomic alteration could provide more information than the bare information of methylation in CIMP grouping.

## DISCUSSION

It has been decades since the first study put forward the idea of CIMP, a subgroup exhibiting hypermethylation of several CGIs in colorectal cancers. Many studies have found this unique cancer subtype in other malignancies, including breast, glioblastoma, lung, stomach and renal carcinoma [7, 8, 27–29]. Those CIMP types in cancers have different influences on clinical performance, and are associated with a series of known classic tumor subtypes. As for the CIMP research in breast cancer, only several studies hinted its relevance to some conventional histological and intrinsic subtypes.

In early times, candidate genes were picked out from a set of known genes related to certain cancer types, and tumor samples were evaluated by the methylation status of those fixed markers. After the high-throughput technologies emerged, whole-genome analysis helped a lot to the molecular classification of breast cancer subtypes, and markers in CIMP characterization got more specific, which were the most typical genes that filtered by statistical methods [27]. However, this approach still couldn't solve the problem of the heterogeneity existed in large sample cohorts, especially when those data were not retrieved from the same batch of experiment. Meanwhile, even we know that the CIMP does occur in many cancers, due to the multiple analytical methods and standards to select markers, how to divide CIMP-H and CIMP-L remains elusive with inconsistency [30].

The lack of enough integration between genetic and epigenetic abnormalities can be a possible reason that caused the confusion of CIMP stratification. Traditionally in colorectal cancers, the influence of CIMP is checked with microsatellite instability (MSI), a chromosomal instability that characterizes the tumor progression. However, heterogeneous cancer subtypes are assessed only by a set of common MSI loci, so its power to evaluate the specific genetic abnormality of tumors can be weakened. Copy number alteration (CNA) is another type of genetic instability in shorter segments than MSI and happens more frequently than MSI. Based on the difference of MSI and CNA, we hypothesize that the specific genes with CNA can also reveal specific genetic variations which occurred in CIMP subtypes when the MSI status can't be retrieved or can't offer adequate information of genomic alteration. Therefore, we tried to find the genes with not only hypermethylation but also recurrent CNA and use them to categorize samples into different copy number and methylation status, where different thresholds were taken. When it comes to genomic information integration, the technical variations would increase inevitably and thus lead to imprecise results. In order to settle this problem, we acquired the methylation and CNA information from the same set of HM450K bead array data, which not only detected epigenomic changes on methylome but also outperformed the CGH arrays in scanning the copy number changes occurred in single genes and avoided unnecessary heterogeneity introduced by batch effects when data were merged.

Moreover, some of markers were related to cancer progression, like IGF2, PTGS2, WNT3A, according to our literature review [31–33]. In CN loss genes, GATA4 is related to the progression of breast carcinoma from early stages and is an independent prognostic factor for survival in breast cancer-specific disease-free IDC patients. Its dispersion can induce distant metastasis, histological grade and HER2 status, but could reduce progesterone receptor in IDC [34]. As for CN gain genes, WNT3A was a highly-expressed gene in ER+ breast cancer cell lines. The expression of PTGS2 (COX2) can influence the physical properties of the tumor microenvironment including human breast cancer cells through mechanotransduction [35]. The IGF2 genetic variants can influence the death risks of breast cancers with BRCA1/2 mutations [36]. Also, Yagi et al. also utilized the SLC30A10 gene to evaluate the methylation status of colorectal cancers, indicating unbiased aberrations in methylation patterns across tumors [37]. In all, those genes tend to show highly cancer-related and can offer a concise view of CNA and methylation status for our samples.

The distribution of the breast cancer luminal A phenotype in CIMP-L tended to differ between CnGain and CnLoss samples. Such enrichment may due to the large portion of luminal A samples, so more information related to those molecular phenotypes may need to be added in further analysis to check out the tendency in this study. Particularly, the CnGain samples retained more CIMP-H samples than CnLoss, implying that copy number gain might correlate with hypermethylation in our markers. In all, the combination of methylation and CNA may shed lights on mechanisms of CIMP and facilitate more accurate cancer risk assessment, detection and outcome prediction.

Current studies related to CIMP still couldn't yield a consistent criterion to partition CIMP-H/L. More specific benchmark is needed for the discovery of CIMP-specific genes and evaluation of sample methylation status for comprehensive understanding of methylation patterns. Inclusion of predetermined CIMP cohorts by high throughput sequencing and clinical with larger dataset would also be suggested as a standard to assess the new CIMP classification methods. Expression mode of markers may also work to reflect the potential mechanisms that regulated by methylation and copy number.

## MATERIALS AND METHODS

### Sample filtering and methylation profiling

Breast adenocarcinoma data of 861 samples (female) was retrieved from TCGA and obtained with the Infinium HumanMethylation450 (HM450K) BeadChips including 485,577 CpG sites with the coverage of 27,176 CGIs and 21231 RefGenes, about 17.2 probes per gene [38].

Methylation profiles were processed by the utilization of the Chip Analysis Methylation Pipeline (ChAMP, vesion 1.4.1), an R package which integrates popular methods to normalize and process the raw .IDAT files from HM450K chips [39]. This 450K pipeline is able to find out differentially methylated genes (DMGs) and detect segments with copy number aberration of samples in a same dataset. The quality control method called subset-quantile within array normalization (SWAN) screened the fraction of failed probes per sample which didn't pass the statistical test [40]. After normalization, “ComBat” method was used to correct batch effects related to slides (Sentrix ID) [41]. To check the efficiency of ComBat normalization, singular value decomposition method (SVD) was used to analyze the biological and technical variations before and after ComBat, and the significant *p values* were presented in heatmaps (Supplementary Figure 4) [42]. All the parameters of functions we used were set as default.

A series of filtering steps were included to pick out suitable samples and probes for our analysis: (1) One sample were removed to meet the slide number standard demanded by ComBat that every slide must have at least two samples for normalization; (2) After removing 17 samples whose failed probe fraction was larger than 0.05, the quality control step was rerun; (3) 69059 probes with a detection *p value* above 0.01 in more than one sample or with a beadcount less than 3 in at least 5% of samples were removed from the analysis; (4) The probes in CGIs were retrieved to generate the methylation profile.

### CIMP marker selection

We used “champ.lasso” in ChAMP to get the differentially methylated probes. The probes in CGIs were picked out with Benjamini–Hochberg adjusted *p* value 0.001, and their differences of DNA methylation level were more than 0.1 between cancers and normal samples. Then the coefficients of variation (CV) in tumors was used to assess the variant extent of those probes, the 50% most variant probes were remained, CV = SD/AVE. Then those probes were matched to their uniquely mapped genes, which were recognized as DMGs hereafter. The methylation value of a gene was calculated as the average beta values of its picked-out probes.

To detect the genes with recurrent CNAs, we mainly integrated different functions in three R packages. Firstly, “ champ.CNA” in ChAMP pipeline was used to get segments with CNAs and their segment mean in every cancer. The mean log2-ratio (segment mean) of a region accessed its copy number status, the cut-off value to call gain and loss of a segment was set as (0.2 and −0.2), and for the CNA evaluation of genes, we chose more strict thresholds to enhance the classification power, which is 0.3 and −0.2 in subsequent analysis. Then the “CNTools” (version 1.22.0) transformed the segment information into a reduced segment matrix (segments as rows and samples as columns), enabling a convenient calculation of the CNA frequency of regions in all samples. Lastly, the R function “cghMCR” (version 1.26.0) converted the matrix of regions into gene copy number profile and calculated their frequencies of CNA in all samples. We found there were apparently more CN gain than CN loss genes according to their distribution of frequency plot (Figure 1C), so 35% and 20% were used as the recurrence percent in patients separately to determine whether a gene's copy number is recurrently gain or loss in tumors.

The overlap part of DMGs and genes of recurrent CNA were selected as markers to evaluate the copy number stage of samples and CIMP status.

### Copy number alteration analysis and CIMP stratification

The copy number status of samples were analyzed by all 25 makers first and then CnGain and CnLoss samples were stratified into CIMP-H/L by 15 markers with copy number gain and 10 markers with copy number loss separately (Supplementary Figure 3). We found that the copy number status of some samples not only presented highly aberrant gains of markers, but also losses. So four CNA stages of samples (CnNone, CnGain, CnLoss, CnBoth) were defined according to the copy number status of 25 genes selected. Since we found the regions of copy number gain is wider and their average segment mean was larger than those of copy number loss, the gene was regarded as CN (+) when its segment mean value was larger than 0.3, and CN (−) when segment mean was less than −0.2. If a sample had at least 6 CN (+) genes in all 15 CN gain genes, it was categorized into CnGain status, and at least 3 CN (−) genes in 10 for CnLoss status. The samples in both CnGain and CnLoss status were categorized into CnBoth status. The left ones were in CnNone status.

The methylation values of 15 CN gain and 10 CN loss markers were used to classify CIMP status of CnGain and CnLoss samples. Different threshold, 0.45 and 0.6 (larger than the 3rd quantile) were used to evaluate the methylation status of CN gain and CN loss genes, separately. If the methylation value was larger than the threshold, the gene was marked as Methylation (+) (Me (+)). CIMP-H tumors were defined as having at least 9 Me (+) in 15 CN gain genes for CnGain samples, and at least 3 Me (+) genes in 10 CN loss genes for CnLoss samples.

### Statistical analysis

CIMP-H/L classification only using methylation information was achieved with hierarchical clustering of methylome characterized by the 601 most variant probes (Ward-D2, Euclidean distance). The datasets were divided into two equal parts for training and validation. The Survival analysis employing the Kaplan–Meier product limit estimator and log rank tests were performed to compare the likelihood of disease-related death of different CIMP cohorts. The multivariate Cox proportional hazard models were fitted to check the survival risks of CIMP types, and covariates included age, tumor stage, etc. Student's *t-test* was used to compare the distribution of methylation levels between copy number gain and copy number loss genes. Chi-square (Fisher test when sample number less than 5) was used to check the distribution difference of molecular phenotypes in CIMP-H/L samples in CnGain/Loss status. All statistical tests were two sided and plots were performed by R program (version 3.2.2).

## SUPPLEMENTARY MATERIALS TABLES AND FIGURES





## References

[1] Ferlay J, Soerjomataram I, Ervik M, Dikshit R, Eser S, Mathers C, Rebelo M, Parkin DM, Forman D, Bray F (2013). GLOBOCAN 2012 v1.0, Cancer Incidence and Mortality Worldwide: IARC CancerBase No. 11.

[2] Li CI, Uribe DJ, Daling JR (2005). Clinical characteristics of different histologic types of breast cancer. Brit J Cancer.

[3] Jones PA, Baylin SB (2002). The fundamental role of epigenetic events in cancer. Nat Rev Genet.

[4] Stirzaker C, Zotenko E, Song JZ, Qu W, Nair SS, Locke WJ, Stone A, Armstong NJ, Robinson MD, Dobrovic A, Avery-Kiejda KA, Peters KM, French JD (2015). Methylome sequencing in triple-negative breast cancer reveals distinct methylation clusters with prognostic value. Nat Commun.

[5] Esteller M (2002). CpG island hypermethylation and tumor suppressor genes: a booming present, a brighter future. Oncogene.

[6] Fang F, Turcan S, Rimner A, Kaufman A, Giri D, Morris LG, Shen R, Seshan V, Mo Q, Heguy A, Baylin SB, Ahuja N, Viale A (2011). Breast cancer methylomes establish an epigenomic foundation for metastasis. Science Transl Med.

[7] Noushmehr H, Weisenberger DJ, Diefes K, Phillips HS, Pujara K, Berman BP, Pan F, Pelloski CE, Sulman EP, Bhat KP, Verhaak RG, Hoadley KA, Hayes DN (2010). Identification of a CpG Island Methylator Phenotype that Defines a Distinct Subgroup of Glioma. Cancer Cell.

[8] Sánchez-Vega F, Gotea V, Margolin G, Elnitski L (2015). Pan-cancer stratification of solid human epithelial tumors and cancer cell lines reveals commonalities and tissue-specific features of the CpG island methylator phenotype. Epigenet Chromatin.

[9] Weisenberger DJ, Siegmund KD, Campan M, Young J, Long TI, Faasse MA, Kang GH, Widschwendter M, Weener D, Buchanan D, Koh H, Simms L, Barker M (2006). CpG island methylator phenotype underlies sporadic microsatellite instability and is tightly associated with BRAF mutation in colorectal cancer. Nat Genet.

[10] Bediaga NG, Acha-Sagredo A, Guerra I, Viguri A, Albaina C, Ruiz Diaz I, Rezola R, Alberdi MJ, Dopazo J, Montaner D, de Renobales M, Fernández AF, Field JK (2010). DNA methylation epigenotypes in breast cancer molecular subtypes. Breast Cancer Res.

[11] Szyf M (2012). DNA methylation signatures for breast cancer classification and prognosis. Genome Med.

[12] Ogino S, Nosho K, Kirkner GJ, Kawasaki T, Meyerhardt JA, Loda M, Giovannucci EL, Fuchs CS (2009). CpG island methylator phenotype, microsatellite instability, BRAF mutation and clinical outcome in colon cancer. Gut.

[13] Lee C, Scherer SW (2010). The clinical context of copy number variation in the human genome. Expert Rev Mol Med.

[14] Krijgsman O, Israeli D, van Essen HF, Eijk PP, Berens ML, Mellink CH, Nieuwint AW, Weiss MM, Steenbergen RD, Meijer GA, Ylstra B (2012). Detection limits of DNA copy number alterations in heterogeneous cell populations. Cell Oncol.

[15] MacDonald JR, Ziman R, Yuen RK, Feuk L, Scherer SW (2014). The Database of Genomic Variants: a curated collection of structural variation in the human genome. Nucleic Acids Res.

[16] Tabarestani S, Ghaderian SM, Rezvani H, Mirfakhraie R, Ebrahimi A, Attarian H, Rafat J, Ghadyani M, Alavi HA, Kamalian N, Rakhsha A, Azargashb E (2014). Prognostic and predictive value of copy number alterations in invasive breast cancer as determined by multiplex ligation-dependent probe amplification. Cell Oncol.

[17] Conway K, Edmiston SN, May R, Kuan P, Chu H, Bryant C, Tse CK, Swift-Scanlan T, Geradts J, Troester MA, Millikan RC (2014). DNA methylation profiling in the Carolina Breast Cancer Study defines cancer subclasses differing in clinicopathologic characteristics and survival. Breast Cancer Res.

[18] Roessler J, Ammerpohl O, Gutwein J, Steinemann D, Schlegelberger B, Weyer V, Sariyar M, Geffers R, Arnold N, Schmutzler R, Bartram CR, Heinrich T, Abbas M (2015). The CpG island methylator phenotype in breast cancer is associated with the lobular subtype. Epigenomics.

[19] Kristensen VN, Vaske CJ, Ursini-Siegel J, Van Loo P, Nordgard SH, Sachidanandam R, Sørlie T, Wärnberg F, Haakensen VD, Helland Å, Naume B, Perou CM, Haussler D (2012). Integrated molecular profiles of invasive breast tumors and ductal carcinoma in situ (DCIS) reveal differential vascular and interleukin signaling. Proc Natl Acad Sci USA.

[20] Feber A, Guilhamon P, Lechner M, Fenton T, Wilson GA, Thirlwell C, Morris TJ, Flanagan AM, Teschendorff AE, Kelly JD, Beck S (2014). Using high-density DNA methylation arrays to profile copy number alterations. Genome Biol.

[21] Nishizaki T, Chew K, Chu L, Isola J, Kallioniemi A, Weidner N, Waldman FM (1997). Genetic alterations in lobular breast cancer by comparative genomic hybridization. Int J Cancer.

[22] Forozan F, Mahlamäki EH, Monni O, Chen Y, Veldman R, Jiang Y, Gooden GC, Ethier SP, Kallioniemi A, Kallioniemi OP (2000). Comparative genomic hybridization analysis of 38 breast cancer cell lines: a basis for interpreting complementary DNA microarray data. Cancer Res.

[23] Courjal F, Theillet C (1997). Comparative genomic hybridization analysis of breast tumors with predetermined profiles of DNA amplification. Cancer Res.

[24] Roylance R, Gorman P, Papior T, Wan YL, Ives M, Watson JE, Collins C, Wortham N, Langford C, Fiegler H, Carter N, Gillett C, Sasieni P (2006). A comprehensive study of chromosome 16q in invasive ductal and lobular breast carcinoma using array CGH. Oncogene.

[25] Kampstra P (2008). Beanplot: A boxplot alternative for visual comparison of distributions. J Stat Softw.

[26] Cancer Genome Atlas N (2012). Comprehensive molecular portraits of human breast tumours. Nature.

[27] Arai E, Chiku S, Mori T, Gotoh M, Nakagawa T, Fujimoto H, Kanai Y (2012). Single-CpG-resolution methylome analysis identifies clinicopathologically aggressive CpG island methylator phenotype clear cell renal cell carcinomas. Carcinogenesis.

[28] Suzuki M, Shigematsu H, Iizasa T, Hiroshima K (2006). Exclusive mutation in epidermal growth factor receptor gene, HER 2, and KRAS, and synchronous methylation of nonsmall cell lung cancer. Cancer.

[29] Hughes LA, Melotte V, de Schrijver J, de Maat M, Smit VT, Bovee JV, French PJ, van den Brandt PA, Schouten LJ, de Meyer T, van Criekinge W, Ahuja N, Herman JG (2013). The CpG island methylator phenotype: what's in a name?. Cancer Res.

[30] Smolich BD, McMahon JA, McMahon AP, Papkoff J (1993). Wnt family proteins are secreted and associated with the cell surface. Mol Biol Cell.

[31] Chen DY, Stern SA, Garcia-Osta A, Saunier-Rebori B, Pollonini G, Bambah-Mukku D, Blitzer RD, Alberini CM (2011). A critical role for IGF-II in memory consolidation and enhancement. Nature.

[32] Menter DG, Schilsky RL, Du Bois RN (2010). Cyclooxygenase-2 and cancer treatment: understanding the risk should be worth the reward. Clin Cancer Res.

[33] Takagi K, Moriguchi T, Miki Y, Nakamura Y, Watanabe M, Ishida T, Yamamoto M, Sasano H, Suzuki T (2014). GATA4 immunolocalization in breast carcinoma as a potent prognostic predictor. Cancer science.

[34] Yoon AR, Stasinopoulos I, Kim JH, Yong HM, Kilic O, Wirtz D, Bhujwalla ZM, An SS (2015). COX-2 dependent regulation of mechanotransduction in human breast cancer cells. Cancer Biol Ther.

[35] Neuhausen SL, Brummel S, Ding YC, Steele L, Nathanson KL, Domchek S, Rebbeck TR, Singer CF, Pfeiler G, Lynch HT, Garber JE, Couch F, Weitzel JN (2011). Genetic variation in IGF2 and HTRA1 and breast cancer risk among BRCA1 and BRCA2 carriers. Cancer Epidem Biomar.

[36] Yagi K, Akagi K, Hayashi H, Nagae G, Tsuji S, Isagawa T, Midorikawa Y, Nishimura Y, Sakamoto H, Seto Y, Aburatani H, Kaneda A (2010). Three DNA methylation epigenotypes in human colorectal cancer. Clin Cancer Res.

[37] Bibikova M, Barnes B, Tsan C, Ho V, Klotzle B, Le JM, Delano D, Zhang L, Schroth GP, Gunderson KL, Fan JB, Shen R (2011). High density DNA methylation array with single CpG site resolution. Genomics.

[38] Morris TJ, Butcher LM, Feber A, Teschendorff AE, Chakravarthy AR, Wojdacz TK, Beck S (2013). ChAMP: 450k Chip Analysis Methylation Pipeline. Bioinformatics.

[39] Hicks SC, Irizarry RA (2015). quantro: a data-driven approach to guide the choice of an appropriate normalization method. Genome Biol.

[40] Johnson WE, Li C, Rabinovic A (2007). Adjusting batch effects in microarray expression data using empirical Bayes methods. Biostatistics.

[41] Teschendorff AE, Zhuang J, Widschwendter M (2011). Independent surrogate variable analysis to deconvolve confounding factors in large-scale microarray profiling studies. Bioinformatics.

