# Supplementary material for: Survival differences of CIMP subtypes integrated with CNA information in human breast cancer
Source: Oncotarget. 2017 Mar 14;8(30):48807–19. doi: 10.18632/oncotarget.16178 (PMC5564726; doi:10.18632/oncotarget.16178)
Supplement: Supplementary file 1 [file oncotarget-08-48807-s001.pdf]

Survival differences of CIMP subtypes integrated with CNA information in human breast cancer

SUPPLEMENTARY MATERIALS

Supplementary Table 1: The probe information of 25 markers. See Supplementary_Table_1

Supplementary Table 2: The clinical characteristics of CIMP-H/L in CnGain/Loss samples

Variables	gain H	gain L	loss H	loss L	*P*-value
**age**					
year	61.2 (± 9.2)	57.8 (± 14.2)	67.0 (± 13.4)	59.6 (± 11.8)	0.02
**Lymph node**					
Mean (SD)	12.6 (± 9.5)	11.9 (± 10.1)	13.5 (± 9.9)	11.1 (± 8.6)	0.66
Missing	2 (10.0%)	4 (6.7%)	1 (4.0%)	5 (3.9%)	
**Stage**					
Stage I/II	14 (70.0%)	37 (61.7%)	16 (64.0%)	94 (73.4%)	0.59
Stage III	6 (30.0%)	21 (35.0%)	9 (36.0%)	33 (25.8%)	
Stage IV	0 (0.0%)	1 (1.7%)	0 (0.0%)	0 (0.0%)	
Stage X	0 (0.0%)	1 (1.7%)	0 (0.0%)	1 (0.8%)	
**Menopause**					
Interminate	0 (0.0%)	2 (3.3%)	0 (0.0%)	5 (3.9%)	0.47
Peri	2 (10.0%)	2 (3.3%)	0 (0.0%)	3 (2.3%)	
Post	14 (70.0%)	38 (63.3%)	19 (76.0%)	90 (70.3%)	
Pre	1 (5.0%)	12 (20.0%)	2 (8.0%)	24 (18.8%)	
Missing	3 (15.0%)	6 (10.0%)	4 (16.0%)	6 (4.7%)	
**Margin**					
Close	0 (0.0%)	1 (1.7%)	0 (0.0%)	3 (2.3%)	1
Negative	18 (90.0%)	50 (83.3%)	21 (84.0%)	107 (83.6%)	
Positive	1 (5.0%)	5 (8.3%)	2 (8.0%)	11 (8.6%)	
Missing	1 (5.0%)	4 (6.7%)	2 (8.0%)	7 (5.5%)	
**Histological type**					
Infiltrating Ductal Carcinoma	16 (80.0%)	44 (73.3%)	18 (72.0%)	81 (63.3%)	0.9
Infiltrating Lobular Carcinoma	3 (15.0%)	10 (16.7%)	4 (16.0%)	32 (25.0%)	
Mixed histology	0 (0.0%)	1 (1.7%)	1 (4.0%)	5 (3.9%)	
Other	1 (5.0%)	5 (8.3%)	2 (8.0%)	10 (7.8%)	
**PAM50**					
Basal-like	0 (0.0%)	8 (13.3%)	1 (4.0%)	4 (3.1%)	0.0006
HER2-enriched	0 (0.0%)	3 (5.0%)	0 (0.0%)	2 (1.6%)	
Luminal A	2 (10.0%)	5 (8.3%)	1 (4.0%)	23 (18.0%)	
Luminal B	3 (15.0%)	2 (3.3%)	7 (28.0%)	6 (4.7%)	
Normal-like	0 (0.0%)	1 (1.7%)	0 (0.0%)	1 (0.8%)	
Missing	15 (75.0%)	41 (68.3%)	16 (64.0%)	92 (71.9%)	

All continuous values are reported with mean and standard deviation, x (± SD), while categories are reported in percentages, no (%).

Supplementary Table 3: The distribution of breast cancer molecular subtypes in CIMP-H/L with different copy number status

Variables	Basal-like	HER2-enriched	Luminal A	Luminal B	Normal-like
Gain/CIMP-H	0 (0.0%)	0 (0.0%)	2 (6.5%)	3 (16.7%)	0 (0.0%)
Gain/CIMP-L	8 (61.5%)	3 (60.0%)	5 (16.1%)	2 (11.1%)	1 (50.0%)
Loss/CIMP-H	1 (7.7%)	0 (0.0%)	1 (3.2%)	7 (38.9%)	0 (0.0%)
Loss/CIMP-L	4 (30.8%)	2 (40.0%)	23 (74.2%)	6 (33.3%)	1 (50.0%)

Supplementary Table 4: The univariate and multivariate Cox proportional hazard regression of CIMP-H/L and other clinical factors in validation set

Clinical factors	Univariate		Multivariate	
	Hazard ratio (95% CI)	*P* value	Hazard ratio (95% CI)	*P* value
**Age(continuos)**	1.033 (0.972–1.097)	0.2941	1.035 (0.971–1.104)	0.2911
**Lymph nodes**	1.029 (0.941–1.125)	0.5341	1.065 (0.926–1.226)	0.3752
**Stage**				
Stage I/II				
Stage III	12.806 (1.149–142.677)	0.0382	52.389 (1.223–2244.692)	0.0389
Stage IV				
Stage X	32.594 (1.477–719.057)	0.0273	718.517 (3.191–1.6E5.538)	0.0173
**Histological type**				
Infiltrating Ductal				
Infiltrating Lobular	0 (0-Inf)	0.9986		
Mixed histology	0 (0-Inf)	0.9998		
Other	0.619 (0.067–5.68)	0.6715		
**Menopause**				
Interminate				
Peri	NA (NA-NA)			
Post	1.136 (0.118–10.945)	0.9121		
Pre	0 (0-Inf)	0.999		
**CIMP in CnLoss**				
CIMP-L/CIMP-H	0.199 (0.034–1.151)	0.0714	0.112 (0.01–1.312)	0.0812

Menopause and histological type are not calculated in multivariate regression because the samples of limited number would yield inaccurate estimation in validation set.

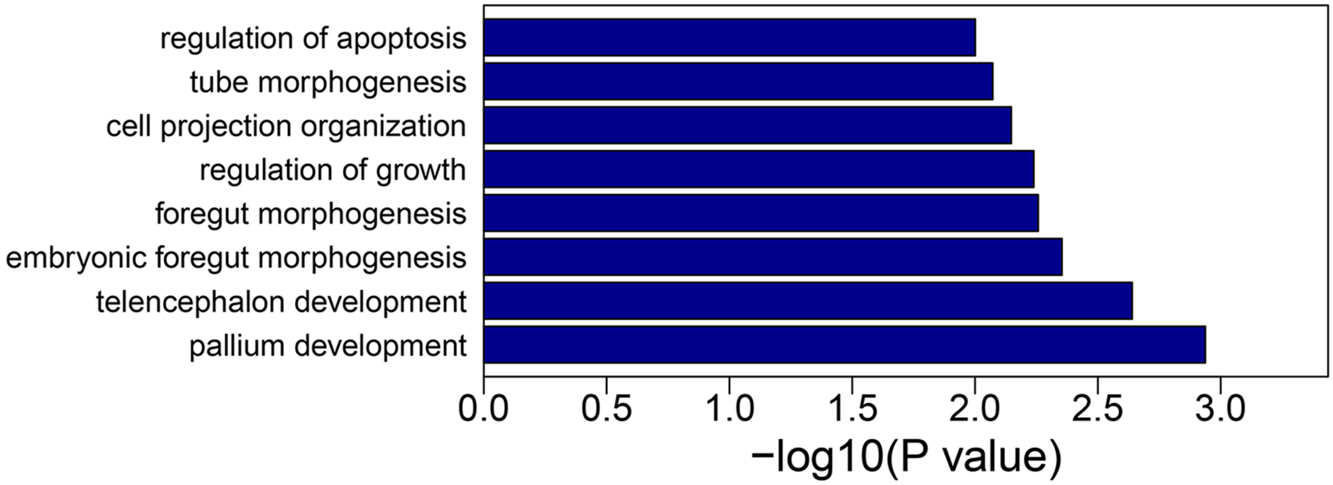

Supplementary Figure 1: The BP GO terms enrichment graph of markers. Most functions were related to cell regulation, adhesion and morphogenesis.

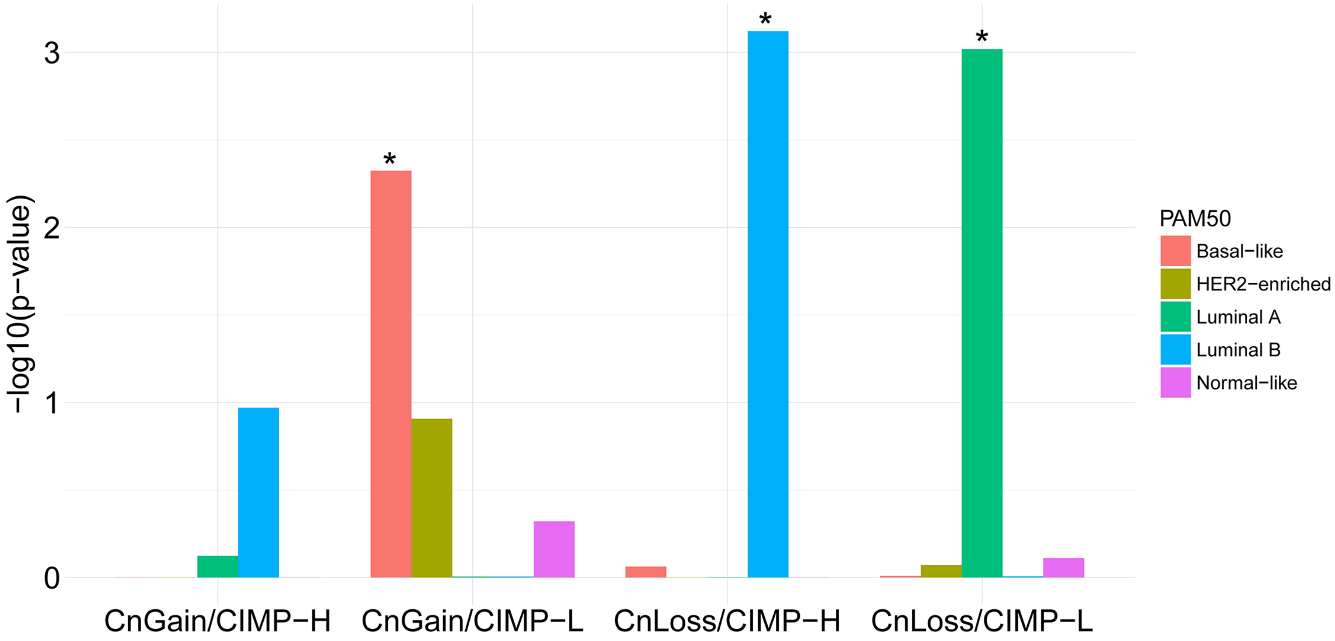

Supplementary Figure 2: The frequency distribution of Me (+) genes in CnGain/Loss samples. The samples were divided into training and validation set equally. (**A**) Histograms of frequency distribution for the Me (+) markers with CN gain in CnGain samples. (**B**) Histograms of frequency distribution for the Me (+) markers with CN loss in CnLoss samples.

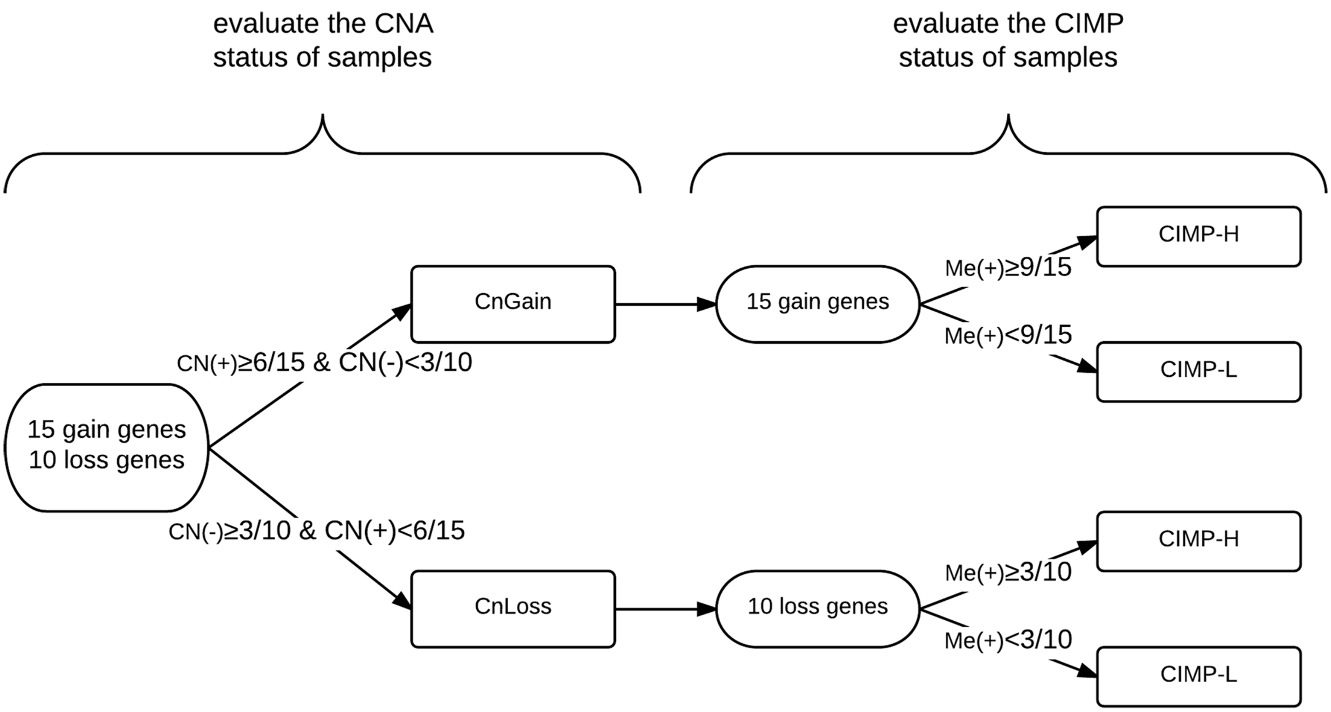

Supplementary Figure 3: The flowchart of CNA and CIMP classification. The copy number statuses of samples were evaluated first, and then the CIMP were classified in CnGain/Loss samples separately by markers with copy number gain or copy number loss.

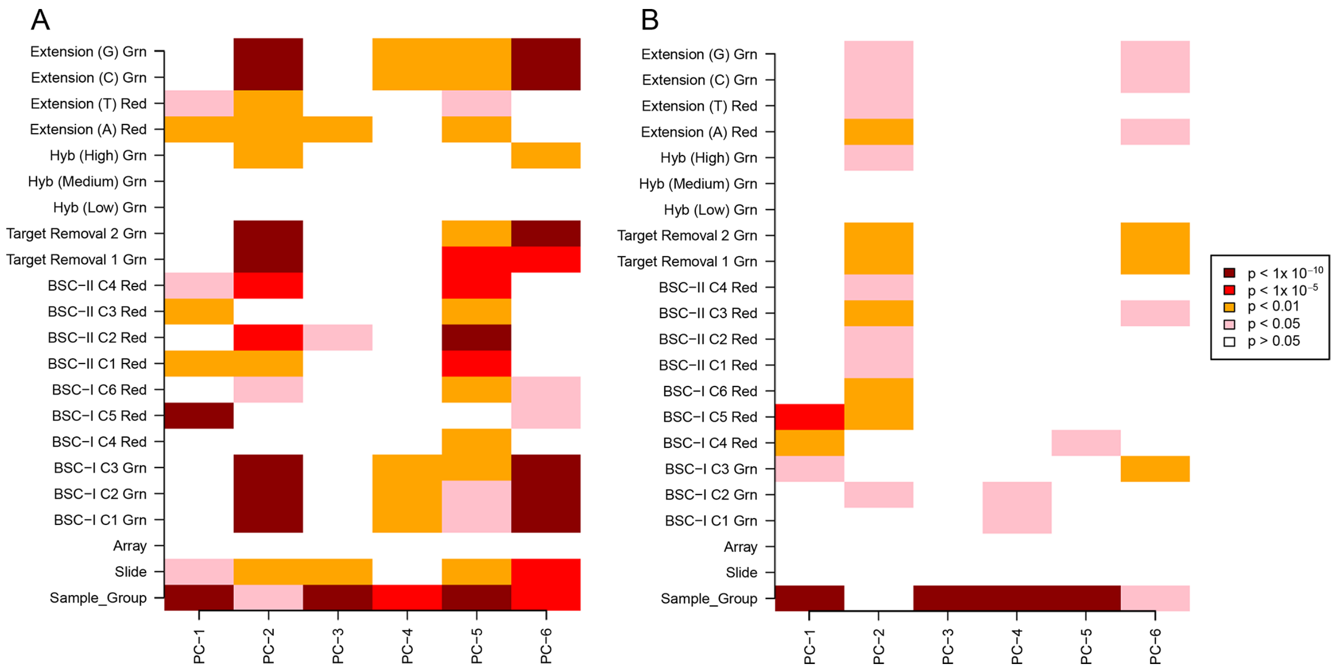

Supplementary Figure 4: The heat map of variation of factors before and after ComBat.
