# Supplementary material for: Survival differences of CIMP subtypes integrated with CNA information in human breast cancer
Source: Oncotarget. 2017 Mar 14;8(30):48807–19. doi: 10.18632/oncotarget.16178 (PMC5564726; doi:10.18632/oncotarget.16178)
Supplement: Supplementary file 2 [file oncotarget-08-48807-s002.docx]

**Supplementary Table 1: The probe information of 25 markers.**

| probeID | adj.P.Val | gene | feature | deltaBeta |
| --- | --- | --- | --- | --- |
| cg09873164 | 1.86E-18 | CRCT1 | Body | 0.2201772 |
| cg16899920 | 8.04E-12 | TDRD10 | TSS200 | 0.10233144 |
| cg02856953 | 1.35E-09 | TDRD10 | TSS200 | 0.11891294 |
| cg17716664 | 4.30E-11 | LMX1A | Body | 0.13112563 |
| cg21868774 | 6.36E-09 | FAM78B | Body | 0.11720903 |
| cg16596102 | 3.83E-17 | FAM78B | Body | 0.15623698 |
| cg02229993 | 1.38E-10 | FAM78B | Body | 0.114847 |
| cg19864490 | 2.96E-14 | FAM78B | TSS1500 | 0.15315301 |
| cg13986130 | 4.29E-20 | PTGS2 | Body | 0.23810339 |
| cg00690431 | 1.41E-19 | PTGS2 | Body | 0.17566238 |
| cg18335243 | 3.07E-11 | PTGS2 | 5'UTR | 0.12949048 |
| cg08482694 | 1.35E-16 | PTGS2 | 5'UTR | 0.19533134 |
| cg17419623 | 7.69E-15 | PTGS2 | TSS200 | 0.17534947 |
| cg05752786 | 6.02E-14 | SYT2 | TSS1500 | 0.10920011 |
| cg17721710 | 3.17E-22 | SLC30A10 | 1stExon | 0.28089547 |
| cg24396691 | 3.13E-22 | SLC30A10 | 1stExon | 0.16177281 |
| cg23815582 | 1.73E-19 | SLC30A10 | 5'UTR | 0.18220798 |
| cg25317664 | 5.02E-18 | SLC30A10 | 5'UTR | 0.17117026 |
| cg24405179 | 3.03E-11 | MIXL1 | TSS200 | 0.11998514 |
| cg22789900 | 2.64E-15 | MIXL1 | 1stExon | 0.17456325 |
| cg06188229 | 1.83E-14 | MIXL1 | Body | 0.13243378 |
| cg15147516 | 5.67E-16 | MIXL1 | Body | 0.16030157 |
| cg09227621 | 5.38E-10 | WNT3A | Body | 0.12497123 |
| cg00078968 | 6.00E-09 | WNT3A | Body | 0.13368275 |
| cg15471815 | 6.89E-07 | HIST3H2BB | TSS1500 | 0.12713428 |
| cg00948275 | 7.03E-08 | HIST3H2BB | TSS1500 | 0.1078584 |
| cg13907959 | 2.31E-08 | HIST3H2BB | TSS1500 | 0.10578878 |
| cg07479670 | 4.04E-15 | NID1 | Body | 0.17690755 |
| cg18879590 | 3.64E-10 | GPR137B | Body | 0.12025838 |
| cg24843474 | 2.37E-16 | RGS7 | TSS200 | 0.20263448 |
| cg23054189 | 4.74E-10 | TRIM58 | 1stExon | 0.15474895 |
| cg20146541 | 9.72E-09 | TRIM58 | 1stExon | 0.13309053 |
| cg06333058 | 7.10E-23 | CLIP4 | TSS1500 | 0.22269809 |
| cg23428985 | 3.18E-23 | CLIP4 | TSS200 | 0.27285635 |
| cg04454951 | 2.88E-19 | VWC2 | TSS1500 | 0.19571529 |
| cg04904331 | 1.83E-18 | VWC2 | TSS1500 | 0.22701895 |
| cg10451078 | 1.53E-12 | GATA4 | TSS1500 | 0.15445096 |
| cg18087943 | 3.29E-11 | INS-IGF2 | Body | 0.13282928 |
| cg17037101 | 3.47E-15 | INS-IGF2 | Body | 0.13996562 |
| cg05743734 | 1.31E-07 | MT1E | 1stExon | 0.11018695 |
| cg04180299 | 5.48E-11 | RLTPR | TSS1500 | 0.13475775 |
| cg03314195 | 8.71E-13 | RLTPR | TSS1500 | 0.13270043 |
| cg00846114 | 3.87E-11 | RLTPR | TSS200 | 0.14123844 |
| cg01762827 | 1.53E-08 | HAS3 | 5'UTR | 0.10182245 |
| cg08747377 | 2.09E-18 | CDH13 | 1stExon | 0.17893742 |
| cg25971347 | 4.32E-17 | FOXF1 | 1stExon | 0.18245948 |
